# Antimicrobial stewardship in pediatrics: focusing on the challenges clinicians face

**DOI:** 10.1186/1471-2431-14-212

**Published:** 2014-08-27

**Authors:** Jennifer Bowes, Abdool S Yasseen III, Nicholas Barrowman, Barbara Murchison, Judy Dennis, Katherine A Moreau, Nisha Varughese, Nicole Le Saux

**Affiliations:** 1 Children’s Hospital of Eastern Ontario Research Institute, 401 Smyth Road, Ottawa, ON K1H 8 L1, Canada; 2 Children’s Hospital of Eastern Ontario, 401 Smyth Road, Ottawa, ON K1H 8 L1, Canada

**Keywords:** Antimicrobial stewardship, Pediatrics, Knowledge

## Abstract

**Background:**

Antimicrobial use is very common in hospitalized children. An assessment of clinician’s prevailing knowledge and clinical approach to prescribing antimicrobials is helpful in order to develop the best strategies for successful stewardship programs. The objectives of the study were to determine fundamental knowledge of principles, approach to antimicrobial use through the clinical vignettes and to identify perceived challenges in decreasing antimicrobial use.

**Methods:**

A questionnaire was developed by subject matter experts and pretested to ensure validity. Using a cross-sectional prospective design, the questionnaire was completed anonymously by staff and trainee physicians at a single tertiary care pediatric hospital between late November 2011 and February 2012.

**Results:**

Of 159 eligible physicians, 86 (54.1%) responded, of which 77 (46 staff and 31 trainees) reported regularly prescribing antimicrobials. The majority of physicians had modest knowledge of factors that would increase risk of resistance however, less than 20% had correct knowledge of local resistance patterns for common bacteria. Almost half of physicians correctly answered the clinical vignettes. Over half of trainees and one third of staff relied most on online manuals for information regarding antimicrobials to assist prescription decision-making. Overall, physicians perceived that discontinuing empiric antimicrobials was the most difficult to achieve to decrease antibiotic use.

**Conclusions:**

Our results highlight several challenges that pediatric practioners face with respect to knowledge and approach to antimicrobial prescribing. Pediatric stewardship programs could in this setting focus on discontinuing antimicrobials appropriately and promoting local antibiograms in the proper clinical setting to decrease overall use of antimicrobials.

## Background

Resistance to nearly all antimicrobial classes is increasing worldwide and threatens patient outcomes [1]. Among patient safety initiatives are antimicrobial stewardship programs (ASPs) whose goals are to promote the optimal use of antimicrobials [2].

Up to half of antimicrobials prescribed in hospital have been deemed unnecessary or incorrect [3,4]. It has been reported that the average number of children in hospital settings who receive at least one antimicrobial is between 33% and 72% [5-8]. A 2009 Canadian point prevalence study reported that 40.1% of pediatric patients in 30 hospitals received antimicrobials [9]. Although most of the evidence supporting antimicrobial stewardship guidelines is derived from adult data, the Infectious Diseases Society of America has identified ASPs in pediatric populations as a research priority.

A questionnaire for trainees and staff was developed to assess knowledge related to principles of stewardship and antimicrobial prescribing practices in a pediatric hospital. The goal was to identify specific challenges that would be helpful in developing targeted ASP initiatives tailored to pediatric needs.

## Methods

### Setting

This was a prospective cross-sectional study, based on an anonymous questionnaire for staff and resident physicians in the Department of Pediatrics at the Children’s Hospital of Eastern Ontario, a tertiary care pediatric center in Ottawa, Canada.

### Survey instrument and administration

A draft questionnaire was developed by an Infectious Disease physician (N.L.S) in conjunction with a questionnaire methodologist (K.M.). Three demographic questions were included to determine respondents’ characteristics, whether they prescribed antimicrobials and the frequency of daily prescriptions.

The goal of the questionnaire was to assess the following dimensions with respect to antimicrobial stewardship: physicians’ education, their knowledge of bacterial resistance and stewardship, their application of principles of antimicrobial stewardship to clinical cases and factors influencing their prescribing habits. Items were then generated for each of these dimensions.

In order to assess the education dimension, two questions focused on their formal education concerning prescribing of antimicrobials. The dimensions of knowledge of stewardship principles and bacterial resistance were assessed with a series of 11 questions pertaining to bacterial resistance and antimicrobial use in the pediatric hospital setting.

In order to operationalize the application of principles and knowledge, five case vignettes with 8 closed ended questions focusing on hospital based care were developed. The vignettes featured common scenarios (peri-operative prophylaxis, lobar pneumonia, urinary tract infection, perforated appendix, non-infectious post-surgery foreign body). These tested the knowledge of surgical prophylaxis (antimicrobial type and length), need for prophylaxis with a chest tube, current Canadian recommendations for treatment of community acquired pneumonia, local recommendations for narrowing the spectrum of antimicrobials according to antimicrobial susceptibility for an uncomplicated pyelonephritis due to *Escherichia coli*, and local empiric therapy regimen recommended for appendicitis.

In order to assess the dimension of influences on prescribing of antimicrobials, one question focused on who they usually relied on for advice on antimicrobial prescribing. Two other questions delved into what aspects of antimicrobial management that would have the greatest impact on decreasing antimicrobial use and their perceived difficulty.

The survey questionnaire was pretested to ensure validity using four different techniques. First, the antimicrobial stewardship literature was reviewed to identify dimensions and items for the questionnaire. Once the questionnaire was constructed based on the items, two individuals from the CHEO Research Institute who are not content experts, reviewed the questionnaire for face validity. Third, two Infectious Disease physicians reviewed the survey for content validity. This was done by asking them to determine if the questions met the requirements for the four above-mentioned dimensions. They were also asked to comment on the clarity and appropriateness of the items and suggest revisions if required*.* Lastly, the questionnaire was piloted with four pediatric physicians, and based on the feedback from these reviews, the questionnaire was revised. The pediatric physicians were excluded from participating in the subsequent survey.

From a list of all staff physicians, the questionnaire (Additional file 1) was distributed electronically from January to February 2012. They were asked to complete a hard copy without identifiers and return it using intra-hospital mail. Staff physicians were asked to send a separate email correspondence to the research assistant to acknowledge completion of the questionnaire to maintain anonymity and ensure no duplicate responses. For residents, the questionnaire was distributed during several academic teaching sessions between November and December 2011. The research assistant was present, checked the name off a trainee list and gathered the completed questionnaires on-site. The questionnaire was re-distributed electronically a month later to non-respondents, and then a hard copy of the survey was mailed to those that had not yet acknowledged having completed the questionnaire. This study was approved by the Children’s Hospital of Eastern Ontario Research Ethics Board. The return of the completed questionnaire implied participant’s consent to participate in the study. Once the survey was closed, answers to the survey were sent electronically to all staff and residents (Additional file 2).

### Statistical methods

Demographics were represented using frequencies and percentages across the identified characteristics, separated by staff physician and trainee. The assessment of physician knowledge on antimicrobial resistance (11 points) and physician knowledge on clinical vignettes (8 points) were computed as the proportion of questions correctly answered to the total number of questions answered. Weighted means and standard deviations of the proportions correct were computed, weighted by the number of questions attempted within the questionnaire. *P* values for the associated relationships of variables across staff and resident physician groups were quantified using the weighted student’s *t* test for knowledge and clinical vignette score comparisons, and Fisher’s exact test otherwise. All data processing and analysis were conducted with R statistical software version 3.0.1 R Development Core Team, 2013, with statistical significance evaluated using two sided p-value at a 5% level of significance [10].

## Results

### Demographics

Surveys were sent to 159 eligible physicians (101 staff physicians and 58 resident trainees). Of the 88 who completed the survey, 2 medical students (who attended the resident teaching sessions) were excluded leaving a total of 86 physicians for a response rate of 54.1%. Of these, nine staff physicians reported that they did not prescribe antimicrobials and were excluded from the analysis. Of the remaining 77, 46 (59.7%) were staff physicians and 31 (40.3%) were resident trainees. All trainees were junior or senior residents in general pediatrics. Of the 46 staff, 19 (41.3%) worked mainly in the outpatient or emergency department, 13 (28.3%) in inpatient general or subspecialty pediatrics, 9 (19.6%) in neonatal or pediatric intensive care units, 4 (8.7%) in surgery, 1 (2.2%) in psychiatry. A total of 23 (74.2%) trainees reported that they had received some formal education on antimicrobial prescribing in the prior year, which was significantly more than the 8 (17.4%) reported by staff, whereas, the majority of staff and trainees reported having had no formal education on antimicrobial stewardship (Table 1). Overall, 40 (51.9%) physicians reported prescribing antimicrobials on average 2 or more times per day.

**Table 1:** Characteristics of antimicrobial prescribing practices and knowledge scores

	**Staff physician n = 46 (%)**	**Resident physician n = 31 (%)**
**Frequency of daily antimicrobial prescribing**		
0-1 times/day	24 (52.2)	13 (41.9)
2-4 times/day	6 (13.0)	15 (48.4)
>4 times/day	16 (34.8)	3 (9.7)
**Formal education of antimicrobial prescribing in past year**		
NO	38 (82.6)	8 (25.8)
YES	8 (17.4)	23 (74.2)
**Formal education on antimicrobial stewardship in past year**		
NO	43 (93.5)	25 (80.7)
YES	3 (6.5)	6 (19.4)
**Resources in making antimicrobial decisions**		
CHEO/HSC manual/Lexicomp/Sanford guide/Red Book	15 (32.6)	18 (58.1)
Pharmacist	12 (26.1)	4 (12.9)
Staff/Peer recommendation/Senior resident recommendation	14 (30.4)	9 (29.0)
Other	5 (10.9)	0 (0.0)
**Parameter with greatest impact in decreasing antimicrobial use**^**∫**^		
Decreasing the length of antimicrobial therapy	2 (4.4)	4 (12.9)
Discontinuing antimicrobials if there is no documented infection	28 (60.9)	11 (35.5)
Early conversion from intravenous to oral therapy	3 (6.5)	1 (3.2)
Narrow spectrum antibiotics versus broad spectrum antibiotics	9 (19.6)	15 (48.4)
**Antimicrobial stewardship parameter most difficult to achieve**^**≠**^		
Decreasing the length of antimicrobial therapy	9 (20.9)	6 (19.4)
Discontinuing antimicrobials if there is no documented infection	21 (48.8)	9 (29.0)
Early conversion from intravenous to oral therapy	5 (11.6)	5 (16.1)
Narrow spectrum antibiotics versus broad spectrum antibiotics	8 (18.6)	11 (35.5)
Overall Percent correct on antimicrobial resistance knowledge scores (mean ± sd)*	57.5 ± 16.6	43.8 ± 12.6
Percent correct on clinical vignettes questions (mean ± sd)*	46.3 ± 21.3	43.9 ± 13.2

*Estimates provided are of the weighted mean and standard deviation, weighted by the number of questions attempted within the questionnaire. P value for group comparisons were computed using the weighted students’ t test.^**∫**^4 staff physicians did not answer this question, ^**≠**^3 physicians did not answer this question.

### Knowledge of and perception of bacterial resistance

Overall, 49 (63.6%) reported having treated patients who were infected with a resistant bacteria (extended spectrum beta-lactamase producing gram negatives, methicillin-resistant *Staphylococcus aureus* or penicillin resistant *Streptococcus pneumoniae*) in the previous year. The number of physicians with correct answers on specific knowledge questions concerning increased risk of resistance were as follows: lower doses versus higher doses, 67 (91.8%) [11], longer courses versus shorter courses 41 (53.9%); piperacillin versus ampicillin 48 (67.6%); ceftriaxone versus gentamicin 53 (75.7%); azithromycin versus clarithromycin 51 (72.9%) [12]. Two (2.6%) physicians correctly identified local prevalence of resistance of *Staphylococcus aureus* to clindamycin, 13 (17.1%) correctly identified local prevalence of penicillin resistance to *Streptococcus pneumoniae*, and 10 (13.2%) correctly identified the local *E. coli* resistance to gentamicin. Third generation cephalosporins were identified as a major risk factor for *Clostridium difficile* colitis by 19 (24.7%) responders. The number of physicians who correctly identified that gut flora would likely be altered after 3 days of antimicrobials was 36 (46.8%). Use of antimicrobials in humans was correctly identified as the greatest risk factor for promotion of resistance by 61 (81.3%) responders. Overall knowledge scores for antimicrobial resistance were 43.8% for trainees and 57.5% for staff.

### Knowledge and application of principles of antimicrobial resistance

Clinical vignette knowledge scores were 43.9% for trainees and 46.3% for staff. The percentage of correct answers was not statistically significantly different between trainees and staff physicians on clinical vignette knowledge scores (p = 0.4110). When grouped by a physician’s reported daily antimicrobial prescribing practices, the knowledge or clinical vignette scores between those who prescribed antimicrobials 0–1 times per day compared to those who prescribed greater than twice a day were not significantly different (p = 0.6736).

### Influences on prescribing habits

When prescribing antimicrobials, trainees reported using published or online manuals as resources more often than staff did (58.1% versus 32.6%; p value = 0.035). Local bacterial resistance rates or resistance information was reported to be considered by 58 (75.3%) of respondents prior to prescribing an antibiotic. Twenty-six (57.8%) staff and 26 (83.9%) trainees felt that the infectious disease service served as a role model for stewardship.

Overall, 39 (50.6%) physicians indicated that discontinuing antimicrobials when there is no documented infection would have the greatest impact on decreasing antimicrobial use. However, 24 (31.2%) indicated that choosing a narrow versus broad spectrum antibiotic would have the greatest impact. More staff physicians compared to trainees felt that discontinuing antimicrobials would have the greatest impact. Discontinuing antimicrobials when there is no documented infection was perceived to be most difficult by staff whereas trainees were more broadly divided among the choices (Table 1).

## Discussion

Few studies have specifically assessed pediatric trainees and staff physician’s knowledge of and clinical approach to common scenarios that illustrate contemporary principles of antimicrobial stewardship. This study provides a perspective of challenges in stewardship in a pediatric setting.

Diagnostic uncertainty, especially in pediatrics when viral infections and non-specific syndromic presentations are more common than in adult medicine, can be a key driver for the use and misuse of antimicrobials, especially when bacterial cultures are negative [13]. In this study, the staff physicians indicated that although discontinuation would have the greatest impact in decreasing days of therapy, discontinuation was also the most difficult to achieve. This is congruent with a study in a neonatal unit where inappropriate use was most commonly due to unnecessary prolongation of therapy [14]. Interestingly, almost half of trainees indicated that narrowing the spectrum would also have an impact on decreasing antimicrobial use and reported that both discontinuing and narrowing the spectrum are difficult to achieve. Although resource manuals are cited as being a frequent resource used, guidance as to discontinuing antimicrobials is neither well enunciated nor described in such manuals. This suggests that reliance on experience and clinical knowledge of disease evolution through a dedicated stewardship program will likely be needed to impact such changes in practice [15,16].

In this study, general knowledge questions scores concerning risk factors for resistance had a wide range of between 53% and 91% correct responses. Lower knowledge scores of 28% to 48% have been previously documented and in at least two studies, were believed to be the primary reason for antimicrobial misuse [16-19].

Physicians scored lower (2.6% to 17.1%) on knowledge of local resistance patterns and often over-estimated the level of resistance compared to the hospital antibiogram. In areas where resistance rates for pediatric pathogens are lower than for corresponding adult groups, this knowledge is potentially critical to prevent escalation to unnecessarily broad spectrum agents, especially empirically. In our setting the published antibiogram resistance rate for *S. pneumoniae* to penicillin is <1% suggesting that other than meningitis or life-threatening infections, penicillins would be reasonable empiric therapy. The inherent limitations of using an antibiogram in individual patients should be balanced however with the individual risk factors of potentially harboring a more resistant pathogen. Ensuring that the local antibiograms are easily accessible is a practical way to have local resistance patterns used more often in clinical decision making particularly for antibiotic naïve patients or those with non-life threatening infections such as urinary tract infections [20].

Clinical vignettes may be valuable in identifying physicians or hospitals where antimicrobial prescribing requires intervention [21,22]. The vignettes we designed were very straightforward and based on published local and national guidelines. Staff and trainees did not differ on their mean percent correct knowledge scores for clinical vignettes despite the fact that staff physicians were less likely to have received any specific education with respect to antimicrobial prescribing. This likely reflects the general knowledge of staff gained through years of experience in treating patients. Perhaps vignettes with more equipoise with respect to stopping antimicrobials would be more revealing as to differences in approach [23].

The main limitations to this study are that the questionnaire was administered exclusively at the one pediatric tertiary care center, and that there was a modest survey response rate. Thus, the results may not be representative of other health care centers and we are unable to account for the contribution of those physicians that did not respond. The non-responders may have come from specialties who do not regularly prescribe antimicrobials but we could not rule in or out the possibility that these groups of physicians are qualitatively different from those included within our study population.

## Conclusions

In pediatric settings, factors such as diagnostic uncertainty play an important role in starting empiric antimicrobials. Challenges identified include improving knowledge of the local antibiogram and focusing on discontinuation of antimicrobials.

## Competing interests

The authors declare that they have no competing interests.

## Author’s contributions

JB participated in the coordination of the study and helped to draft the manuscript. AY performed the statistical analysis. NB performed the statistical analysis. BM participated in the design of the study. JD participated in the design of the study. KM participated in the design of the study. NV participated in the design of the study. NLS conceived of the study, and participated in its design and coordination and helped to draft the manuscript. All authors read and approved the final manuscript.

## Pre-publication history

The pre-publication history for this paper can be accessed here:

http://www.biomedcentral.com/1471-2431/14/212/prepub

## Supplementary Material

Additional file 1 Antimicrobial Use Knowledge and Attitudes Survey. Click here for file

Additional file 2 Answers: Antimicrobial Use Knowledge and Attitudes Survey. Click here for file
