# Supplementary material for: Antimicrobial stewardship in pediatrics: focusing on the challenges clinicians face
Source: BMC Pediatr. 2014 Aug 27;14:212. doi: 10.1186/1471-2431-14-212 (PMC4236642; doi:10.1186/1471-2431-14-212)
Supplement: Additional file 1 — Antimicrobial Use Knowledge and Attitudes Survey. [file 1471-2431-14-212-S1.pdf]

Antimicrobial Use Knowledge and Attitudes Survey

We need your help to evaluate antimicrobial stewardship at CHEO. This stewardship involves the optimal use of antimicrobials to improve patient outcomes and minimize harm or side effects. The return of this completed survey will act as your implied consent to your participation.

1. **What is your role at CHEO?**

   - ☐ Staff physician
   - ☐ **Junior** resident in pediatrics (year 1 or 2)
   - ☐ **Senior** resident/**Fellow** in pediatrics (year 3 or 4)
   - ☐ Resident from another service (family medicine etc.)
   - ☐ Medical student
   - ☐ Other, please specify: ______________________

2. **Where do you conduct the majority of your clinical work?**

   - ☐ Emergency
   - ☐ General Pediatrics
   - ☐ PICU
   - ☐ NICU
   - ☐ Outpatient
   - ☐ Oncology
   - ☐ Surgery
   - ☐ Outside of CHEO
   - ☐ Other, please specify _______________________

3. **Do you prescribe antimicrobials?**

   - ☐ Yes
   - ☐ No

4. **In an average day, how many times do you prescribe antibiotics?**

   - ☐ 0-1 times/day
   - ☐ 2-4 times/day
   - ☐ >4 times/day

5. **In the past year, have you had any <u>formal</u> education of antimicrobial <u>prescribing</u> (indication, dosing)?** ***Exclude informal discussion on rounds but include any specific education sessions.***

 Yes, go to question 5a) and 5b)
 No, skip to question 6

5a) IF YES, how many hours of antimicrobial prescribing education have you had in the past year?

 1-4 Hours
 5-7 Hours
 ≥8 Hours

5b) Who taught the education session(s)?

Please check all that apply.

 Infectious Disease Physician
 Pharmacist
 Pediatrician
 Other, please specify ______________________

6. **In the past year, have you had any <u>formal</u> education on antimicrobial <u>stewardship</u> (de-escalation, optimization of dosing, relating to antibiograms)?** ***Exclude informal discussion on rounds but include any specific education sessions.***

 Yes, go to question 6a) and 6b)
 No, skip to question 7

6a) IF YES, how many hours of antimicrobial stewardship education have you had in the past year?

 1-4 Hours
 5-7 Hours
 ≥8 Hours

6b) Who taught the education session(s)?

Please check all that apply.

 Infectious Disease Physician
 Pharmacist
 Pediatrician
 Other, please specify ______________________

7. **Have you treated any patients in the last year that had resistant bacteria such as (ESBL, MRSA or penicillin resistant *Strep. Pneumoniae*)?**

 Yes
 No
 Cannot recall

8. **For each of the following pairs of prescriptions, which contributes MOST to promoting antimicrobial resistance?**

Circle only one of each pair.

a) Lower doses **or** Higher doses

b) Longer courses **or** Shorter courses (≤ 7 days)

c) Ampicillin **or** Piperacillin (for equivalent time)

d) Gentamicin **or** Ceftriaxone (for equivalent time)

e) Azithromycin **or** Clarythromycin (for equivalent time)

9. **In your opinion, how many days of antibiotics does it take to change the flora (bacteria) in the gut and pharynx to more resistant bacteria?**

Please check only one.

 3 days
 6 days
 9 days
 Unsure

10. **According to the CHEO 2010 antibiogram, what is the % resistance that was found for the following bacteria?**

a) Group A streptococcus **resistant to** clindamycin
 1% 3% 5% 10% 20% Unsure

b) Streptococcus pneumonia **resistant to** penicillin
 1% 3% 5% 10% 20% Unsure

c) Escherichia coli (E. coli) **resistant to** gentamicin
 1% 3% 5% 10% 20% Unsure

11. **Which <u>one</u> of the following antibiotics would be MOST likely to increase the risk of development of *Clostridium difficile* infection?**

 cefotaxime
 clindamycin
 piperacillin
 vancomycin
 Unsure

The following are scenarios about principles of antimicrobial stewardship.

12. **You are asked to write antibiotic orders for a teenager who is having a spinal fusion for insertion of spinal rods. What would you likely recommend for the pre-surgical prophylaxis drug and length of antimicrobial post-operatively?**

 Not applicable to my specialty

Antibiotic: ________________________ Unsure

Length of post -operative treatment:
Please check only one.

 24 hours
 48 hours
 72 hours
 Unsure

13. **You are asked on rounds about a child who has had 10 days of antibiotics following resection of a cystic adenomatoid malformation with a chest tube in the pleural space. The new medical student asks if this is appropriate or inappropriate use of antimicrobials. What would your likely response be?**

 Appropriate
 Inappropriate
 Unsure

14. **You are admitting a 3 year old child who has received all recommended immunizations to CHEO. The child had cough, fever and chest pain for 4 days. He has a temperature of 38.5°C, a RR of 30/min, a HR of 90/minute and an oxygen saturation of 98% on room air. His radiograph shows a moderate size RML infiltrate. What would be the most narrow spectrum recommended empiric antimicrobial therapy?**

 Not applicable to my specialty

 cefuroxime
 ampicillin or penicillin
 cefuroxime and clindamycin
 Unsure

14a) The child shows clinical improvement (afebrile after 48 hours and eating) and the blood cultures are negative. What would you likely prescribe as outpatient oral antimicrobial therapy?

- cefuroxime axetil
- amoxicillin
- Clavulin® (amoxillin and clavulinate)
- Unsure

14b) What is the recommended total length of the outpatient prescription assuming no complications?

- 5 days
- 7 days
- 10 days
- Unsure

15. A previously healthy 6 year old girl presents to the Emergency Department with burning upon urination for 2 days. She is febrile and is admitted. The urine analysis has > 50 WBC and after 48 hours, the urine culture has *E. coli* that is susceptible to all cephalosporins. The child is now afebrile, has normal urinary tract anatomy and you would like to send her home to complete the antibiotics. Which antibiotic would be the most appropriate as outpatient oral antimicrobial therapy in this case?

- Not applicable to my specialty

Please check only one.

- oral cefuroxime axetil
- oral cephalexin (Keflex)
- oral cefixime
- Unsure

16. You are asked to write antibiotic orders for a previously well 11 year old child who has clinical appendicitis and is going to the operating room. Which of the following antimicrobials are recommended as first line therapy?

- Not applicable to my specialty

Please check only one.

- cefotaxime and metronidazole
- cefotaxime alone
- gentamicin and clindamycin
- gentamicin and metronidazole
- Unsure

17. Which one of the following activities do you think contributes MOST to bacterial resistance worldwide?

Please check only one.

☐ Use of antibiotics in animals
☐ Use of antibiotics in humans
☐ Transmission of resistant bacteria in hospitals
☐ Other, please specify ____

18. In making antimicrobial decisions on individual patients who or what do you rely on the <u>MOST</u> when you have questions?

Please check only one.

☐ Staff/Peer recommendation / Senior resident recommendation
☐ Pharmacist
☐ CHEO / HSC manual / Lexicomp / Sanford guide / Red Book
☐ Other, please specify ____

19. When prescribing an antibiotic for a patient, which type of bacterial resistance rates or resistance information do you think about before prescribing the antibiotic?

Please check only one.

☐ Local rates
☐ National rates
☐ World reports on resistance
☐ Other, please specify ____
☐ None

20. Which of the following parameters would likely have the <u>GREATEST</u> impact in decreasing antimicrobial use in the unit you work?

Please check only one.

☐ Discontinuing antimicrobials if there is no documented infection
☐ Decreasing the length of antimicrobial therapy
☐ Early conversion from intravenous to oral therapy
☐ Narrow spectrum antibiotics versus broad spectrum antibiotics
☐ Other, please specify ____

21. In terms of influencing what you prescribe, to what extent do you feel the infectious disease service serves as a role model for stewardship on hospital wards?

Please check only one.

☐ Not at all
☐ Some of the time
☐ Most of the time
☐ All the time

22. Which ONE of the following tasks do you consider being MOST DIFFICULT to do with respect to modifying antimicrobial therapy?

Please check only one.

☐ Discontinuing antimicrobials when there is no documented infection
☐ Decreasing the length of antimicrobial therapy
☐ Early conversion from intravenous to oral therapy
☐ Narrow spectrum antibiotics versus broad spectrum antibiotics

Thank you for your participation!
