# Supplementary material for: Antimicrobial stewardship in pediatrics: focusing on the challenges clinicians face
Source: BMC Pediatr. 2014 Aug 27;14:212. doi: 10.1186/1471-2431-14-212 (PMC4236642; doi:10.1186/1471-2431-14-212)
Supplement: Additional file 2 — Answers: Antimicrobial Use Knowledge and Attitudes Survey. [file 1471-2431-14-212-S2.doc]

**Answers: Antimicrobial Use Knowledge and Attitudes Survey**

1. **For each of the following pairs of prescriptions, which contributes MOST to promoting antimicrobial resistance?**

*Circle only one of each pair.*

a) Lower doses **or** Higher doses

Lower doses permit the bacteria that have low level resistance to multiply and increase the chances that they will in turn be resistant.

b) Longer courses **or**  Shorter courses (≤ 7 days)

Longer courses (10 days or more in original studies) exposes the bacteria in the body to antibiotics for longer periods of time thereby promoting the survival of more resistant bacteria.

c) Ampicillin  **or** Piperacillin (for equivalent time)

Since piperacillin is broader spectrum, it will kill off many more susceptible bacteria of many types and leave only the small amount of resistant ones who are then left at the end of therapy to multiply.

d) Gentamicin **or** Ceftriaxone (for equivalent time)

Ceftriaxone has a large spectrum of activity and will leave behind only resistant bacteria to multiply.

e) Azithromycin **or**  Clarithromycin (for equivalent time)

Azithromycin acts intracellularly and therefore stays in the white blood cell. It will therefore ‘elute’ slowly and has a very long half-life of 5 days. It will be present for a prolonged period of time thus permitting bacteria to develop resistance. Just 3 days of azythoromycin can produce *Streptococcus pneumonia* which is resistant.

1. **In your opinion, how many days of antibiotics does it take to change the flora (bacteria) in the gut and pharynx to more resistant bacteria?**

*Please check only one.*

 3 days

 6 days

 9 days

 Unsure

When systemic antibiotics are used, the potential for changing the bacteria in the oropharynx and the gut is large. Thus resistant bacteria can emerge while the patient is being treated for infection. The longer a patient receives antibiotics, the greater potential that the bacteria will change. No one knows for sure in all cases but the studies have shown that this occurs fairly early (~3 days) after starting. Thus to avoid this, stopping antibiotics if one decides there is no infection and maintaining the recommended time for prophylaxis is critical.

1. **According to the CHEO 2010 antibiogram, what is the % resistance that was found for the following bacteria?**

The **antibiogram** **is found on the Infection Prevention and Control site on CHEOnet.** It is a useful measure of the antibiotic resistance profiles of commonly isolated bacteria in the region and is produced yearly by the Microbiology Laboratory at CHEO.

a) Group A streptococcus (GAS) **resistant to** clindamycin

 1%  3%  5%  10%  20%  Unsure

The 2010 antibiogram indicated 3% resistance. According to the 2011 antibiogram (98% of the GAS are susceptible to clindamycin).

b) Streptococcus pneumonia **resistant to** penicillin

 1%  3%  5%  10%  20%  Unsure

The 2010 antibiogram had 3% resistant to penicillin. The current 2011 antibiogram indicates that **100%** of the 59 isolates recovered at CHEO were susceptible to penicillin

c) Escherichia coli (E. coli) **resistant to** gentamicin

 1%  3%  5%  10%  20%  Unsure

The E. coli susceptibility to gentamicin remains at 95%.

1. **Which one of the following antibiotics would be MOST likely to increase the risk of development of *Clostridium difficile* infection?**

 cefotaxime

 clindamycin

 piperacillin

 vancomycin

 Unsure

Although any antimicrobial can increase the risk of C. difficile, within this list, cefotaxime is very broad spectrum and has little activity against anerobic bacteria and therefore is the agent that increases the risk for *Clostridium difficile* the most. Ciprofloxacin use, especially in adults has also been implicated as increasing the risk of *C. difficile* substantially. Using cefotaxime (and other broad spectrum antibiotics) judiciously and limiting the number of days on antibiotics in the hospital will further decrease the occurrence and spread of *C. difficile*.

***The following are scenarios about principles of antimicrobial stewardship.***

1. **You are asked to write antibiotic orders for a teenager who is having a spinal fusion for insertion of spinal rods. What would you likely recommend for the pre-surgical prophylaxis drug and length of antimicrobial post-operatively?**

 Not applicable to my specialty

**Antibiotic**: _cefazolin____________________  Unsure

Length of post -operative treatment:

*Please check only one.*

 24 hours

 48 hours

 72 hours

 Unsure

1. **You are asked on rounds about a child who has had 10 days of antibiotics following resection of a cystic adenomatoid malformation with a chest tube in the pleural space. The new medical student asks if this is appropriate or inappropriate use of antimicrobials. What would your likely response be?**

 Appropriate

 Inappropriate

 Unsure

The recommendation for a non-infected site is no more than 1 or 2 doses of perioperative antibiotics.

1. **You are admitting a 3 year old child who has received all recommended immunizations to CHEO. The child had cough, fever and chest pain for 4 days. He has a temperature of 38.5°C, a RR of 30/min, a HR of 90/minute and an oxygen saturation of 98% on room air. His radiograph shows a moderate size RML infiltrate. What would be the most narrow spectrum recommended empiric antimicrobial therapy?**

 Not applicable to my specialty

 cefuroxime

 ampicillin or penicillin

 cefuroxime and clindamycin

 Unsure

The Canadian and AAP pneumonia guidelines recommend intravenous ampicillin or penicillin for a child who is admitted with a non-complicated pneumonia AND who is not severely ill. This is appropriate since it covers the most common organism likely to be causing the pneumonia ( *Streptococcus pneumonia* and Group A streptococcus).

**14a) The child shows clinical improvement (afebrile after 48 hours and eating) and the blood cultures are negative. What would you likely prescribe as outpatient oral antimicrobial therapy?**

 cefuroxime axetil

 amoxicillin

 Clavulin® (amoxillin and clavulinate)

 Unsure

Amoxicillin is the drug of choice for step-down therapy. Amoxillin/clavulinate would be indicated if one were also targeting *Staphylococcus aureus* sensitive to cloxacillin.

**14b) What is the total recommended length of the antimicrobials for uncomplicated pneumonias in children?**

 5 days

 7 days

 10 days

 Unsure

The recommendation is for about one week of therapy in uncomplicated pneumonia. Given that she has had 2 days of therapy it may be reasonable to prescribe another 5 days to complete a week. The length of therapy also depends on the size of the infiltrate and the rapidity of clinical response.

1. **A previously healthy 6 year old girl presents to the Emergency Department with burning upon urination for 2 days. She is febrile and is admitted. The urine analysis has > 50 WBC and after 48 hours, the urine culture has *E. coli* that is susceptible to all cephalosporins. The child is now afebrile, has normal urinary tract anatomy and you would like to send her home to complete the antibiotics. Which antibiotic would be the most appropriate as outpatient oral antimicrobial therapy in this case?**

 Not applicable to my specialty

*Please check only one.*

 oral cefuroxime axetil

 oral cephalexin (Keflex)

 oral cefixime

 Unsure

Given that it is susceptible to cephalosporin, the one that has the narrowest spectrum would be cephalexin (a first generation cephalosporin).

1. **You are asked to write antibiotic orders for a previously well 11 year old child who has clinical appendicitis and is going to the operating room. Which of the following antimicrobials are recommended as first line therapy?**

 Not applicable to my specialty

*Please check only one.*

 cefotaxime and metronidazole

 cefotaxime alone

 gentamicin and clindamycin

 gentamicin and metronidazole

 Unsure

Gentamicin and metronidazole will be appropriate treatment as pre-operative treatment for appendicitis. If the appendix is ruptured, these can be continued for a time after the surgery.

The use of third generation cephalosporins is discouraged in this scenario as this is considered too broad spectrum for this community-acquired infection and can increase the risk of *C. difficile*.

1. **Which one of the following activities do you think contributes MOST to bacterial resistance worldwide?**

*Please check only one.*

 Use of antibiotics in animals

 Use of antibiotics in humans

 Transmission of resistant bacteria in hospitals

 Other, please specify ___________________________________________

Although important in persons who interact with animals, the use of antibiotics in animals contributes less to the evolving bacterial resistant in humans compared to unrestricted use in humans. The use of broad spectrum antibiotics and globalization of human travel contributes substantially to worldwide resistance patterns. Locally, prevention of transmission through good hand washing and PPE is critical in preventing hospital transmission.

1. **Which of the following parameters would likely have the GREATEST impact in decreasing antimicrobial use in the unit you work?**

*Please check only one.*

 Discontinuing antimicrobials if there is no documented infection

 Decreasing the length of antimicrobial therapy

 Early conversion from intravenous to oral therapy

 Narrow spectrum antibiotics versus broad spectrum antibiotics

 Other, please specify _____________________________________________

All these maneuvers will play important roles in good antimicrobial stewardship. In our survey, both staff and residents felt that discontinuing antimicrobials would have a large impact however felt it was the most difficult thing to do.
